# Spatial Pattern Dynamics of 3D Stem Cell Loss of Pluripotency via Rules-Based Computational Modeling

**DOI:** 10.1371/journal.pcbi.1002952

**Published:** 2013-03-14

**Authors:** Douglas E. White, Melissa A. Kinney, Todd C. McDevitt, Melissa L. Kemp

**Affiliations:** 1 The Wallace H Coulter Department of Biomedical Engineering, Georgia Institute of Technology & Emory University, Atlanta, Georgia, United States of America; 2 The Parker H. Petit Institute for Bioengineering and Bioscience, Georgia Institute of Technology, Atlanta, Georgia, United States of America; Princeton University, United States of America

## Abstract

Pluripotent embryonic stem cells (ESCs) have the unique ability to differentiate into cells from all germ lineages, making them a potentially robust cell source for regenerative medicine therapies, but difficulties in predicting and controlling ESC differentiation currently limit the development of therapies and applications from such cells. A common approach to induce the differentiation of ESCs *in vitro* is via the formation of multicellular aggregates known as embryoid bodies (EBs), yet cell fate specification within EBs is generally considered an ill-defined and poorly controlled process. Thus, the objective of this study was to use rules-based cellular modeling to provide insight into which processes influence initial cell fate transitions in 3-dimensional microenvironments. Mouse embryonic stem cells (D3 cell line) were differentiated to examine the temporal and spatial patterns associated with loss of pluripotency as measured through Oct4 expression. Global properties of the multicellular aggregates were accurately recapitulated by a physics-based aggregation simulation when compared to experimentally measured physical parameters of EBs. Oct4 expression patterns were analyzed by confocal microscopy over time and compared to simulated trajectories of EB patterns. The simulations demonstrated that loss of Oct4 can be modeled as a binary process, and that associated patterns can be explained by a set of simple rules that combine baseline stochasticity with intercellular communication. Competing influences between Oct4+ and Oct4− neighbors result in the observed patterns of pluripotency loss within EBs, establishing the utility of rules-based modeling for hypothesis generation of underlying ESC differentiation processes. Importantly, the results indicate that the rules dominate the emergence of patterns independent of EB structure, size, or cell division. In combination with strategies to engineer cellular microenvironments, this type of modeling approach is a powerful tool to predict stem cell behavior under a number of culture conditions that emulate characteristics of 3D stem cell niches.

## Author Summary

Pluripotent embryonic stem cells can differentiate into all cell types making up the adult body; however, this process occurs in a complex three dimensional environment with many different parameters present that are capable of influencing cell fate decisions. A model that can accurately predict the strengths of factors influencing cell fate would allow examination of spatial and temporal patterns of cell phenotype. For this study, we focused on the earliest fate transition that occurs in 3D clusters of embryonic stem cells by monitoring the presence of a transcription factor (Oct4) associated with stem cell pluripotency. After experimentally classifying patterns that arise en route to a fully differentiated aggregate *in vitro*, we constructed a computational model to deduce how stem cells integrate cues from their surrounding environment to give rise spatial patterns. We used a rules-based modeling approach in which information exchanged by cells with their nearest neighbors regulated cell fate decisions. This parsimonious model captured the spatial dynamics of early cell lineage commitment in a 3D multicellular structure. In combination with strategies to modulate cellular environments, our model provides a flexible tool for elucidating the extra- and intercellular interactions regulating spatially organized differentiation of stem cells in 3D.

## Introduction

Pluripotent embryonic stem cells (ESCs) have the unique ability to differentiate into cells of the three germ lineages that form all of the tissues and organs of a mature organism. Differentiation of pluripotent ESCs can be induced *in vitro* via a variety of existing approaches to emulate aspects of the developmental program. One of the most widely used techniques relies upon the formation of multicellular aggregates composed of undifferentiated ESCs in suspension culture, commonly referred to as embryoid bodies (EBs) [1], [2], that spontaneously induce the differentiation of ESCs within the 3D aggregate [3], [4]. Due to the fact that EBs mimic the physical structure and cellular composition of the morphogenic embryonic microenvironment, they have been used to study aspects of development *in vitro* as well as the formation of primitive tissue complexes [3]–[5]. Despite the utility of the approach, robust methods to control EB differentiation *in vitro* remain limited due to an incomplete understanding of the complex interactions within the 3D multicellular aggregates that mitigate cell fate decision [6], [7]. The development of techniques to control ESC differentiation *in vitro* requires an improved understanding of cellular cues that regulate differentiation and the means to precisely control these complex signals.

Considerable effort has focused on ascertaining the role of individual components of the cellular microenvironment in regulating cell fate decisions. The extent to which cell-cell communication via paracrine [8], [9], autocrine [9]–[11], or direct contact signaling [12]–[14] enhance or inhibit differentiation have been investigated in various contexts. Exogenous manipulation has been used to control differentiation by the addition or removal of various soluble factors in a temporally regulated manner in an effort to mimic morphogenic cues. Factors that preserve pluripotency (e.g. LIF [15]–[17], BMP-4 [15]) and factors that can initiate differentiation (e.g. Activin A [18], FGF-2 [18], and retinoic acid [19]) have been thoroughly examined, both in terms of the appropriate doses and their temporal administration. In many cases, the signaling pathways and modes of action of these growth factors are also known but the effects of combinatorial treatments can be difficult to predict *a priori* [16], [20]. Although exogenous factors have proven necessary for the *in vitro* and *in vivo* maintenance or differentiation of ESC populations, they are not the only factors regulating stem cell behaviors. The biochemical composition of the cellular microenvironment [9], [21] and extracellular matrix (ECM) [22]–[24] have also been implicated in the regulation of cellular niches. In addition, the mechanics and physical properties of the microenvironment can also impact cell phenotype [25]. Given that cell fate transitions occur in complex environments where biochemical and physical cues coexist, elucidating the role each of these combinatorial factors via experimental studies alone remains a significant challenge. Therefore, although the aforementioned studies can provide information about certain individual factors in isolation, new approaches that allow systematic investigation of combinations of parallel factors that regulate stem cell differentiation are needed to more accurately predict cell response to complex microenvironmental cues.

In many instances, computational modeling strategies have been successfully used to recapitulate the integration of complex signals that direct cell fate decisions and correctly predict the resulting phenomena. Depending on the desired resolution of the system, ordinary differential equations can be used to model a variety of processes in stem cells including - but not limited to - cellular signaling events [26]–[28], protein interaction networks [29], and genetic networks [30]. Partial differential equations can be used to assess spatial changes introduced via diffusion of molecules; this approach has been extensively studied to examine gradients of nutrients in cancer cell spheroids [31], as well as mass transfer limitations in EBs [32]. Alternatively, to model the structure of cellular aggregates [33]–[35], cellular division and tissue formation [36]–[43], and pattern formation in biological systems [38], [39], agent-based modeling has been applied to overlay rules-based and physical modeling approaches [34]. Moreover, agent-based models have been used to investigate dynamic processes of multicellular systems, such as morphogenesis [43], [44] and formation of physical tissues [45]. Investigation of the spatial and temporal regulation of stem cell differentiation using agent based model approaches has not been attempted, yet the ability to examine how structural features of the stem cell niche influence the spatial patterns associated with loss of pluripotency is attractive for studying differentiation in 3D EB systems. This study demonstrates the utility of computational rules-based modeling to predict emergent spatial patterns associated with one pluripotent transcription factor in EBs (Oct4) and investigate macroscopic principles that can play important roles in determining cell fate transitions.

## Results

### Modeling Embryoid Body Structure

**Figure 1:**
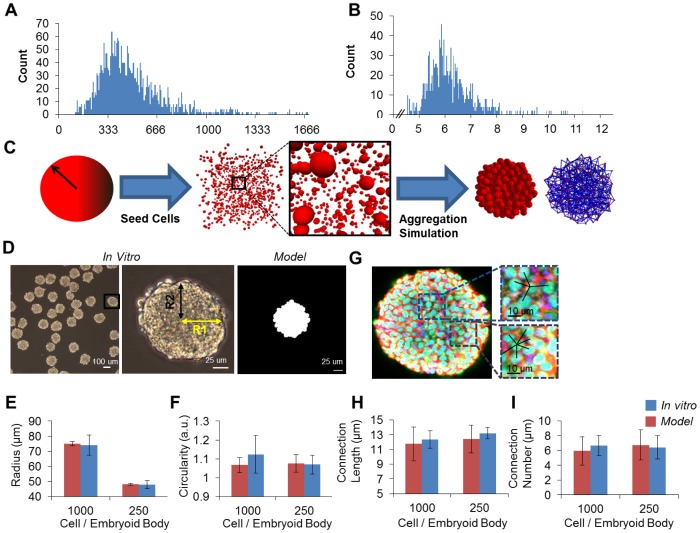
Aggregate modeling methodology. Dissociated mouse embryonic stem cells (mESCs) were analyzed via Coulter counter for surface area (A) and radius (B). *In silico* aggregates were generated in a physical modeling process in which cells were generated and then forcibly aggregated (C). Embryoid bodies (EBs) were formed via ultra-high throughput methods for two initial cell numbers: 250 and 1000 - a representative image for 1000 cell EB is shown (D). The black box in first column is digitally enlarged in the second column. Circularity was calculated by fitting the EB to an ellipse and taking the ratio of the two radii labeled R1 and R2 respectively. EBs were analyzed for two macro scale aggregate properties: radius (E) and circularity (F). Confocal images were used to analyze local aggregate properties –a representative 1000 cell EB image is shown (G). Aggregate local properties were assessed by two metrics: connection length (H) and number of connections (I).

Embryoid bodies (EBs) are 3D multicellular spheroidal aggregates that self-assemble via E-cadherin mediated interactions in suspension conditions [46], [47]. Our first goal in constructing a model description of EBs was to accurately recapitulate the overall multicellular structure based on the physical properties of individual mouse embryonic stem cells (mESCs). Prior models of multicellular structures have described the individual cellular agents as incompressible objects consisting of ellipsoids [48]. We opted to use a physics-based modeling approach in which cells were modeled as incompressible rigid spheres as this is a powerful and portable method for representing complex aggregate shapes. To determine if modeling mESCs as spheres was appropriate, the effective surface area (Fig. 1A) and radii (Fig. 1B) of individual ESCs were experimentally determined via Coulter counter analysis. The average surface area to volume ratio of the mESC line was 3.26+/−0.15, which is only ∼8% higher than the theoretical value of 3.00 for a spheroid. Due to the increased computational costs associated with an ellipsoid collision detection algorithm and the relatively low error in the surface area-to-volume ratio (<10%), we proceeded by representing each cellular agent as a sphere. The distribution of cell radii from the Coulter counter measurements (Fig. 1A, B) were used to create the population of spheres for each agent in the EB simulations. These cell agents were randomly seeded into a box, which served as an initial boundary for the simulation, and then forced to aggregate using a gravitational point source into a multicellular spheroidal structure.

The structures of *in silico* and *in vitro* aggregates were assessed for aggregates of 250 and 1000 cells using four parameters: radius, circularity, connection count, and connection lengths. Size and circularity were used to assess the entire aggregate structure and were experimentally determined through the analysis of phase contrast images, while similar measurements were obtained using projections of the *in silico* EBs onto a 2-dimnesional plane (Fig. 1D). The results indicated that the model appropriately captured the macroscopic features of the relative EB aggregates since there were no statistical differences between the model and experimental metrics (Fig. 1E, 1F). The connection count and connection length parameters were calculated from the spatial distribution of individual cells comprising the aggregates and serve as quantifiable metrics for assessing local micro-scale organization within EBs. These parameters were assessed by individual cell labeling performed in confocal microscopy images and via computational algorithms for the *in silico* EBs (Fig. 1G). As an example, the blue box in Fig. 1G highlights a cell with an average connection length of 14.87+/−2.07 microns and connection number of 4. For *in silico* EBs, the aggregates were “virtually sectioned” (at a 10 µm thickness) to perform similar analysis on a 2D projection, and neither the average circularity nor the connection lengths differed statistically from the experimentally derived EB values (Fig. 1H, 1I). Overall, these results quantitatively comparing four different physical parameters indicated that the model was able to accurately create the structure of individual EBs on both the aggregate and cellular scales, providing an accurate structural framework for our subsequent analysis of spatial patterning.

### Spatial Patterns Associated with Loss of Pluripotency

**Figure 2:**
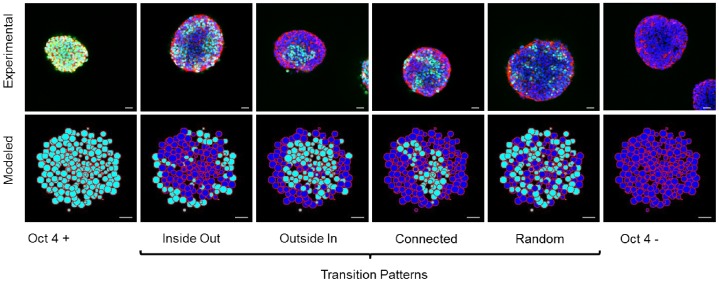
Classification of differentiation spatial patterns within EBs. Six classifications are used to described Oct4 expression patterns: undifferentiated, inside-out, outside-in, connected, random, and differentiated. Confocal images shown on top are stained with DAPI (blue), phalloidin (red) and Oct4 (green) (with a scale bar of 25 µm). The model generates patterns similar to confocal images with Oct4− cells (dark blue) and Oct4+ cells (cyan). Scale bar represents 25 µm.

Throughout the subsequent discussion of the results, pluripotent cells that exhibit loss of Oct4 expression are simply referred to as “differentiated”, acknowledging the caveat that Oct4− cells are not terminally differentiated. As Oct4 is concomitant with loss of pluripotency, it was used to monitor the pluripotent state of the cells [49]–[52]. This process has been modeled as a bi-stable transition, which causes an all or none response [52]. The temporal patterns of loss of pluripotency were evaluated in 250- and 1000-cell EBs via confocal microscopy to examine Oct4 expression. Starting from a homogeneous population of undifferentiated cells, spatial heterogeneity (as defined by loss of Oct4 expression) was observed over the course of the multiple days of evaluation. In order to capture the diversity of spatial pattern heterogeneity, a classification system was developed. Based on preliminary results from both the experimentally derived EBs and the model, six different categories of patterns were proposed: Oct4+, inside-out, outside-in, connected, random, and Oct4− (Fig. 2). These patterns can be loosely grouped into three larger categories: Oct4+, transitioning, and Oct4−. The outside-in, inside-out, connected, and random patterns were all considered transition patterns as they captured intermediate stages of the differentiation process. Inside-out patterns are characterized by differentiation in the middle of the EB and undifferentiated cells on the outside; conversely outside-in patterns exhibit differentiation on the outside, and undifferentiated cells in the middle. Connected patterns were defined as multiple distinct connected regions of cells of the same state, whereas random patterns were classified as no identifiable pattern based on a lack of connectivity.

**Figure 3:**
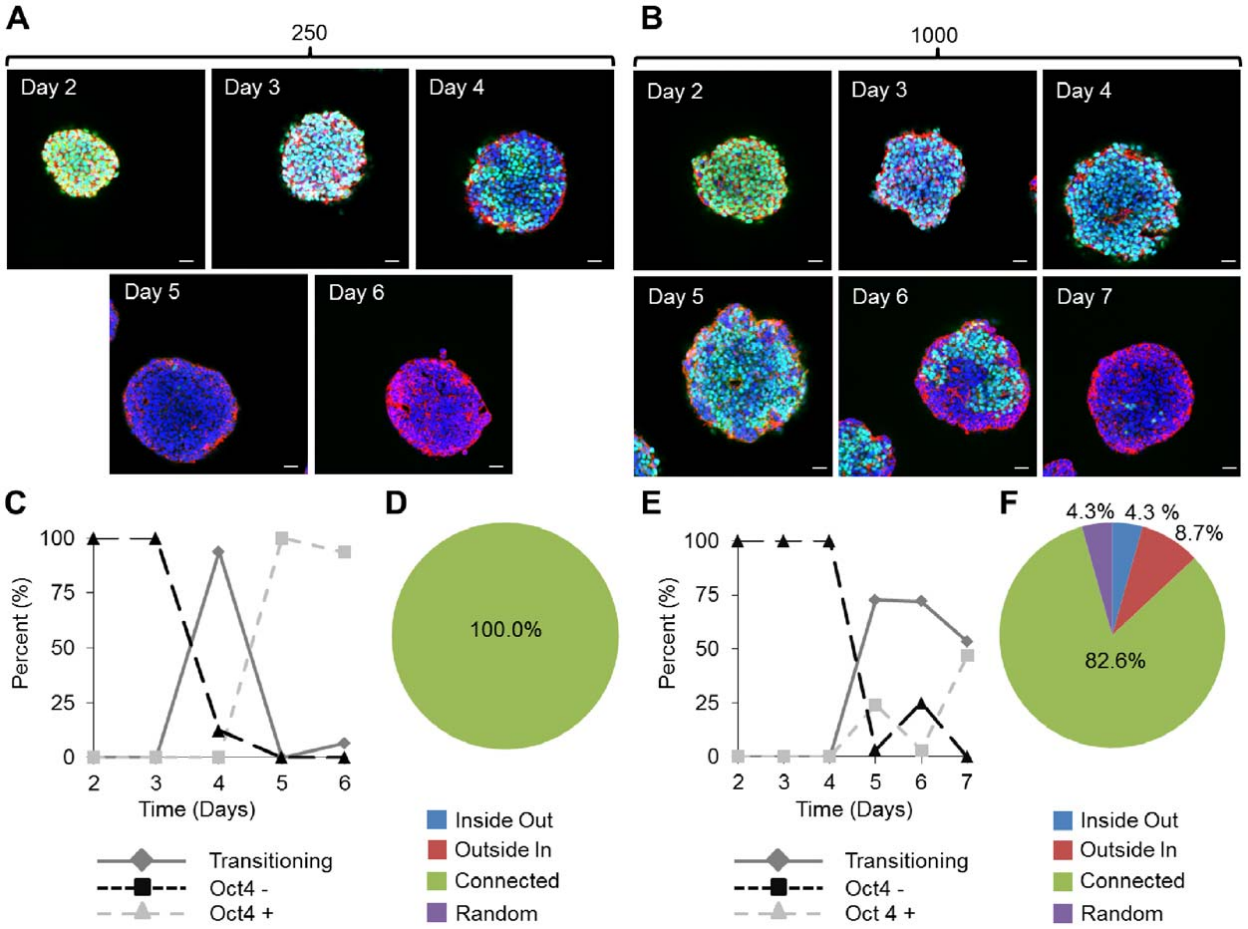
Transition times for differentiation patterns within EBs vary as a function of size. Confocal images of EBs stained with DAPI (blue), phalloidin (red), and Oct4 (green) shown at a depth of 25 µm for EBs of (A) 250 and (B) 1000 cells. (C, E) Temporal dynamics of observed patterns for 250 cell EBs (C) and 1000 cell EBs (E). (D, F) the overall distribution of observed patterns for 250 cell EBs over 6 days in culture (D) and 1000 cell EBs over 7 days in culture (F). Scale bars on all images are 25 µm.

In the smaller 250-cell EBs, Oct4 expression persisted for up to six days (Fig. 3A). Rapid loss of Oct4 was observed between days 3 and 5 (Fig. 3C) and the patterns associated with differentiation were classified entirely as “connected” (Fig. 3D). In 1000-cell EBs, differentiation patterns were assessed over a 7-day period (Fig. 3B). Differentiation was observed to occur at a later time than the smaller 250-cell EBs, with transition patterns occurring from days 4 to 7 (Fig. 3E). The spatial patterns in the 1000-cell EBs associated with differentiation were more varied than the 250-cell EBS but also were primarily classified as “connected” (Fig. 3F). At each time point, pattern classification for each EB size was performed to generate temporal differentiation profiles for each time point (Fig. 3D, 3F). The trajectories of differentiation were calculated by assessing how the number of differentiated, undifferentiated, and transitioning patterns changed over time. Although the types of patterns associated with differentiation only changed slightly with EB size (Fig. 3D, 3F), the kinetics of the process did appear to change appreciably. The 250-cell EBs began differentiating at ∼ day 3 and finished within one day, whereas the 1000-cell EBs started a similar process later at ∼ day 4, and took nearly 3 days to fully exhaust Oct4 expression (Fig. 3C, 3E).

### Static Rules-Based Modeling of the Cellular Microenvironment

**Figure 4:**
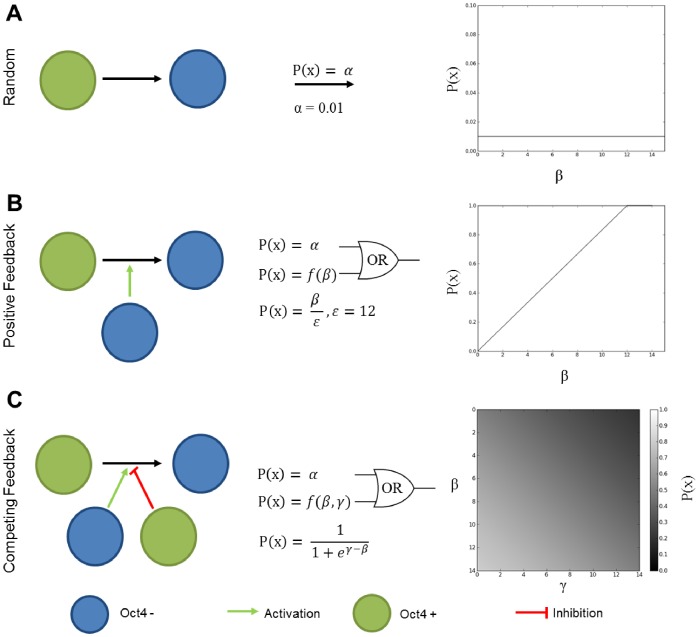
Overview of rule configurations. Three different rule configurations governing transition from the undifferentiated to differentiated state are shown: random (A), positive feedback (B), and competing feedback (C). The random rule is governed by a constant probability of differentiation denoted by α (A). The positive feedback rule takes into account Oct4− cells denoted by β, and allows them to positively influence the differentiation probability (B). The competing feedback rule take into account β and also the number of Oct4+ cells denoted by γ (C). In the case of the positive feedback and competing feedback rules, the probability are combined using an or-gate logical operator. Representation of the probaility density functions P(x) are shown for each rule.

After validating the generation of an appropriate 3D geometry of EB aggregates, rules-based modeling was performed by creating network structures, in which “nodes” represented individual cells, and “connections” represented physical interactions between adjacent cells; nodes were allowed to convey information with the macrostructure along the defined connections. The goal was to determine if simple rules accurately produced the distribution of spatial patterns observed experimentally. During these initial simulations the macro-structures were assumed to be static (i.e. no proliferation, migration or apoptosis). Cells could exist in either of two states: undifferentiated (Oct4+) or differentiated (Oct4−). The transition between these two states was chosen as binary based on previous modeling work [52] and occurred based on different rule formulations: “random”, “positive feedback” or “competing feedback” (Fig. 4). The random rule configuration represented a stochastic, basal differentiation probability (Fig. 4A). The positive feedback rule was based on a paradigm in which differentiated cells bias neighboring cells to differentiate (Fig. 4B) and was inspired by differentiation induced via direct cell-cell interactions [13], [53]. Finally, the competing feedback rule depicts a situation where differentiated cells promote subsequent differentiation of neighboring cells while undifferentiated cells inhibit this transition (Fig. 4C). Positive feedback in this rule was based upon the known role of soluble factors to maintain pluripotency [54], while negative feedback comes from the differentiation induced via the cell-cell interactions discussed above [13], [53].

**Figure 5:**
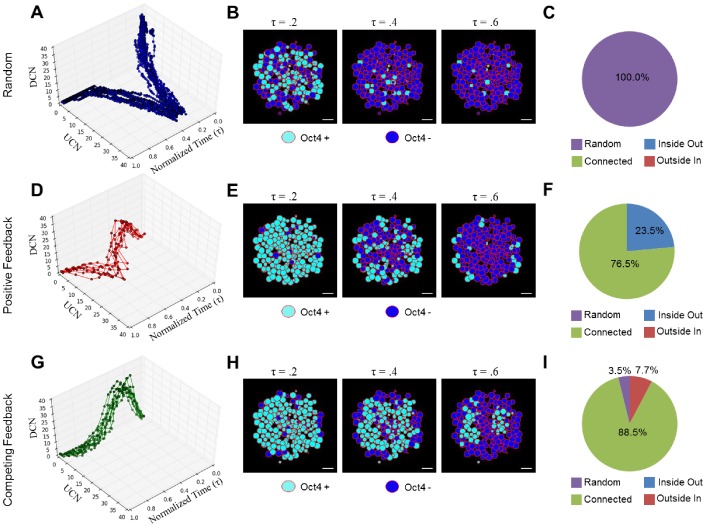
Spatial pattern trajectories of differentiation for 1000 cell aggregates. (A, D, G) Pattern trajectories are shown for all three rules for 1000-cell EBs plotted against a normalized time axis (τ) where the time step was divided by the total number of time steps required for the simulation to complete. (B, E, H) Representative “virtual sections” of aggregates over the course of a simulation. Cyan represents Oct4+ cells while blue signifies Oct4− cells. (C, F, I) Differences in the kinetics of modeled differentiation for all three rules. All scale bars are 25 µm.

Representative outcomes for each of the rules are shown for 250 (Fig. S1) and 1000 cells (Fig. 5) per EB. The patterns represented by different rules did not differ significantly across EB sizes (Fig. 5 and Fig. S2); however, differences in the distribution of the patterns were observed between different rule configurations. The random rule transitioned through largely random patterns (Fig. 5I, S2I), whereas both the positive feedback and the competing feedback exhibited an enrichment in the connected patterns (Fig. 5F, S2F and Fig. 5C, S2C respectively). Because the experimental EBs differentiated largely through connected patterns (Fig. 3D,F) based on the pattern classification, it was difficult to evaluate which rule configuration(s) best emulated the experimental data. To ensure these results were not an artifact of the multicellular aggregate structure, resultant patterns of 250 and 1000 cell EBs were evaluated for each of the three rules (Fig. S2) across numerous different simulated structures without discernible changes in outcome.

### Quantitative Pattern Analysis

To glean insight into the evolution of the connected patterns, we used two quantitative metrics, undifferentiated cluster number (UCN) and differentiated cluster number (DCN), to assess pattern formation and simulation trajectories against a normalized time (τ) axis. Analyzing the cell phenotype transitions by the UCN and DCN metrics revealed distinct paths of pattern formation for each of the different rules. The qualitative shape of the trajectories was independent of EB sizes (Fig. S3). From such curves, critical points (τ = .2, τ = .4, and τ = .6) representing rapid changes or important regions across all rules were chosen and representative EB slices were displayed (Fig. 5B,E,H)). Analysis of the trajectories themselves revealed insight about the types of clusters being formed in the “connected” patterns. In the “positive feedback” scenario, the loss of Oct4 expression was characterized by a high number of differentiated or undifferentiated clusters, suggesting localized intercellular neighbor influences regulating phenotype transition (Fig. 5D, Fig. S1D). The peak in UCN at τ = .6 was characterized by a large number of isolated pockets of undifferentiated cells. In contrast, the “competing feedback” rule peaked through a high number of differentiated clusters, but never amassed a high undifferentiated cluster number (Fig. 5G, Fig. S1G) which matched the larger isolated and persistent clusters of Oct4 positive cells experimentally observed in both the 250- and 1000-cell EBs. Taken together, these data indicate that the “competing feedback” rule matched the patterns observed biologically with the highest fidelity for the different size EBs examined.

Furthermore, the trajectory analysis provided novel information about the evolution of certain patterns. For example, random differentiation can be characterized by a high initial spike in the DCN as this signifies the emergence of several small clusters of differentiated cells (Fig. 5A, Fig. S1A), and the duration of this spike represents how long the random patterns persist throughout the duration of the model. If the UCN remains fairly low, the pattern transitions into a connected phenotype, again evidenced by the low number of undifferentiated clusters of cells (Fig. 5B, Fig. S1B). When the UCN remains at 1, this signifies either an inside-out, or outside-in pattern. However, if the UCN transitions towards a high value, this signifies that the differentiation is still largely governed by random patterns (Fig. 5A, Fig. S1A). Overall, these metrics provide quantitative metrics for assessing the types of patterns formed, and the evolution of these patterns over time. One limitation of the model, however, was that the kinetics of pattern formation could not explain the differences in kinetics experimentally observed between different EB sizes (Fig. 3D,F). This suggested that although a static size aggregate modeling approach was sufficient for predicting the prevalence of different spatial pattern classifications, it did not fully capture the kinetics of experimental Oct4 loss. In order to further investigate the kinetics of the pattern transitions, we modified our modeling framework to include cell division and embryoid body growth.

### Dynamic Rules-Based Modeling

**Figure 6:**
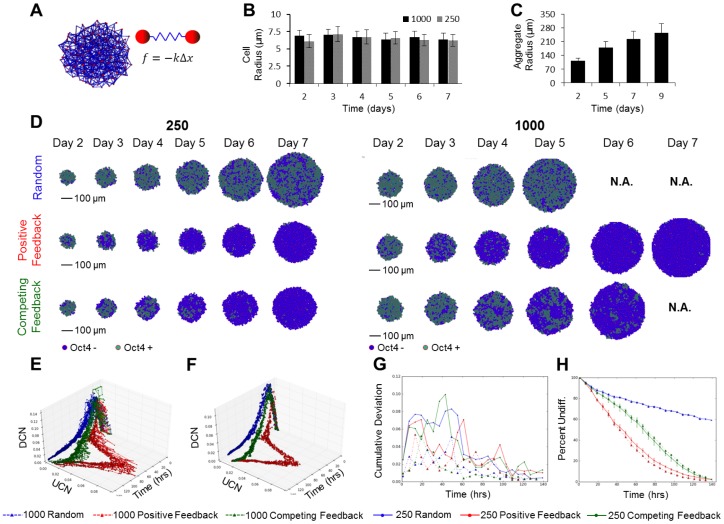
The effects of cell division on spatial pattern trajectories. (A) Mass spring schema for modeling cellular division and adhesion. (B) Coulter coulter data showing the radius of stem cells from embryoid bodies and how they change over the 6 day differentiation time course. (C) Estimated sizes of embryoid bodies used to estimate growth rates. (D) Representative virtual slices of simulated 250 and 1000 cell dividing embryoid bodies for the three different rules. Oct4+ cells are shown in teal and Oct4− cells in blue. UCN, DCN time plot trajecory for the 250 cell EBs (E) and 1000 cell EBs (F). (G) Summation of the variance in the UCN, DCN trajectories. (H) The percent of undifferetiated cells as a function of time.

We hypothesized that dynamic processes, such as cell division and growth of the EB aggregate, influence spatial patterns of Oct4 expression loss. To investigate the effect of cell division on this loss of pluripotency transition, a revised model which could simulate growing EB structures was created in which the Oct4+ cells divided at a faster rate than the Oct4− cells. With this approach, cells were modeled as rigid spheres while cell-cell connections were represented by springs which helped maintain the overall macro-structure of the aggregate (Fig. 6A). We observed experimentally that the size of differentiating ESCs did not change appreciably with time, which reduced complexity from the model description (Fig. 6B). The first step was to determine an estimated cellular division rate for Oct4+ and Oct− cells. This was accomplished using experimental growth data approximated from the size of the embryoid bodies (Fig. 6C), yielding a rate of ∼18 hours for division of our stem cells and ∼51 hours for division of the differentiated cells which is consistent with the literature [55]. Using these division rates, we applied the rules we derived in the former static model to the new dynamic model. As an internal control, we ran division simulations with no rules to monitor any bias introduced by the model (Fig. S4) and found that the cells grew in an exponential manner (Fig. S4A), while the density of the aggregates remained constant (Fig. S4C). Furthermore, the average connection number and connection lengths of the network remained constant with time (Fig. S4 E). Connection length remained constant as a function of the aggregate radius, whereas the connection number decreased on the outer layer of the aggregate, as was expected (Fig. S4E). Taken together these results suggest that structurally no bias was introduced into the model by introducing cell division.

Next we examined the spatial patterns formed under the various rule configurations during enlargement of the EB over a 5–7 day period, with the pattern trajectories now normalized by the cell number to account for cell growth. Trajectories simulated over the 6 day culture period indicated consistent pattern distributions and their evolution over time (Fig. 6E–F). While few differences were observed in the trajectories as a function of EB size, the cumulative variance in the UCN and DCN metrics was greater for the 250 cell EBs (Fig. 6G), suggesting a more heterogeneous population of differentiated cells. At later time points (>Day 5) the positive feedback rule generated clusters of undifferentiated cells predominantly on the outer edges of the EB. In contrast, the competing feedback rule produced larger clusters of undifferentiated cells localized towards the center of the EB. The trajectories of these growth simulations matched the general shape of the trajectories for the static simulations (Fig. S3), with a notable exception of the random rule. Analysis of the percentage of undifferentiated trajectories revealed that over half the cells remained undifferentiated at the seven day time point, which may explain the absence of the DCN to UCN transition observed in the previous static model simulations.

The motivation behind modeling a dynamic EB structure was to more closely recapitulate the emergent morphogenic processes occurring over the transitional 5–7 day period and to investigate whether the inclusion of cell division and EB enlargement influenced the rate of emergent spatial patterns (Fig. 6H). In the case of the random and competing feedback rules, no observable difference in the percentage of differentiated cells appeared, however the positive feedback rule resulted in the percentage of undifferentiated cells being lower in the 1000 cell versus 250 cell EBs, likely due to the total number of cells present. Taken together these results suggest that differential cell division does not significantly influence the formation or evolution of phenotype patterns over time and the pattern formation process is dominated by the regulatory mechanisms encoded in the probabilistic rules.

## Discussion

Differentiation is a complex biological process involving the coordinated regulation of multiple genes by intrinsic and extrinsic factors. Rather than attempt to model the intricate network of genetic circuitry, signaling mechanisms, and environmental cues, we approached the loss of pluripotency from a simplified perspective designed to elucidate the most basic principles dictating pattern formation in a spherical multicellular system. We developed a modeling framework capable of recapitulating the physical properties of embryoid bodies for multiple sizes and under conditions of cell division and aggregate growth. This framework allowed us to simulate how different probabilistic rules were manifest in the emergence of spatial patterns and to examine the evolution of these patterns over time. Through comparison of the simulated pattern trajectories with the pattern classification developed for our experimental data, we determined that all possible pattern classes - as well as a similar distribution within these classes - are possible with the agent rules employed. Furthermore, from both static and dynamic frameworks, our simulations indicate that aggregate structure, size, and growth are physical features that do not dictate the distribution of spatial patterns or their trajectories as a function of time.

Differentiation is classically thought of as a binary transition from the undifferentiated pluripotent stem cell state to differentiated phenotypes in embryoid body (EB) models [5], [31], [46]. Models of stem cell differentiation often consider these events on a population basis [56] or at an intracellular signaling level [57]. Here we have shown that the transition from Oct4+ to Oct4− states produces dynamic, spatially heterogeneous patterns in a continuous manner. These results indicate that modeling embryonic stem cell (ESC) fate decisions as a stochastic, binary process is sufficient to predict the dominant emergent spatial patterns of loss of pluripotency. Additionally, 250- and 1000-cell EBs undergo loss of pluripotency through the same intermediate patterns which suggests that the macro processes governing this early stem cell transition (while occurring at slightly different rates) are largely independent of size. In addition, comparing static structures and cell division models revealed that spatial transition patterns and their evolution are not significantly affected by cell growth.

Our modeling approach created *in silico* aggregates with similar properties to in vitro EBs. The spring-based constraint representing cellular adhesions was able to accurately capture the evolution of the aggregate architecture. Additionally, the high-level rules described here were able to reproduce the emergence of a variety of spatial patterns, all of which could be observed experimentally in EBs. Both modeled and experimental EBs demonstrated enrichment in connected patterns of cells. Quantification of model simulation outputs by differentiated and undifferentiated cluster number (DCN and UCN) allowed visual representation of the time evolution of connected patterns in 3D multicellular aggregate systems. The use of the UCN and DCN metrics provided information about not only the types of connected patterns formed, but also the transitions between the different types of spatial patterns. A combination of analyses using these metrics and manual pattern identification indicated that the “competing feedback” rule scheme that accounted for opposing influences of neighboring pluripotent and differentiated cells produced a distribution of spatial patterns that most closely resembled the experimentally observed spatial patterns for both 250- and 1000-cell EBs. This was observed in both the static and dynamic division models, and could be hypothetically represented biologically as a combination of cell-cell signaling and the complex interplay of local soluble factors and other chemical gradients that influence pluripotent cell fate decisions. [17], [53], [58], [59]. However, this is only one possible explanation for a coarse-grained description, and a variety of other signaling pathways and molecules are likely involved in regulating this transition. Interestingly, none of the rules were able to explain the observed emergent patterns if cells were not allowed to also spontaneously differentiate at a low rate random rate. Although for the positive feedback rule this follows directly from the construction of the probability equations, the formation of strongly connected patterns was also not observed in the competing feedback rule without the inclusion of this low stochastic rate (data not shown), suggesting this random rate is important to capture the experimentally observed transition patterns.

The static structure model predicted small differences in the Oct4 expression kinetics of EBs with different cell seeding numbers should exist; however, the slight changes observed did not capture the full extent of the variation present in the experimental results. The inclusion of cell division (with a faster division rate for pluripotent cells) was also not able to explain the difference in the kinetics of this process. These results indicate that additional factors in the changing culture environment may modulate the kinetics of Oct4 loss in a size-dependent manner; hydrodynamic effects [60], diffusion limitations and/or local chemical gradients may need to be taken into account for changing aggregates sizes [32], [61] in order to reproduce the experimentally observed differences in differentiation kinetics. It is also possible that the rules chosen are not descriptive of our system with a fine enough resolution, thus explaining the ability to explain pattern formation characteristics but not kinetics. Furthermore, we constructed the model such that all cells have the same strength when affecting other cells, which assumes that cells convey the same amount of information regardless of the amount of cell connections or the amount of shared cell area, an assumption that may need to be refined as more detailed information about the nature of intercellular communication is included. Future developments will account for cell migration, local versus distal cell-cell communication and diffusion within the EB to investigate how these traits affect the physical microenvironment. The top-down modeling approach described in this study provides new insight into the spatial pattern development associated with differentiation of ESCs in 3D EB structures. Surprisingly, without explicitly modeling diffusion gradients or specific signal transduction mechanisms, features of temporal and spatial regulation were elucidated. Transitions in cell phenotype can be modeled as a flow of information between cells that is largely based on stochastic fate changes that are influenced by the cells' surrounding environment. The model was able to identify a simple rule paradigm that is biologically relevant and consistent with the current knowledge of stem cell regulatory processes [17], [53], [58], [59]. A comparison of static and division models indicated that the proposed rule schemes are not significantly affected by cell division. Consequently, the proposed modeling technique developed thus far has demonstrated validity for exploring the propensity of various spatial patterns observed in EBs during the differentiation process. This model can readily be extended to investigate what factors influence differentiation into different germ layers, and eventually used to predict optimal niche and microenvironment organization for efficient stem cell maintenance and differentiation.

## Materials and Methods

### Cell Culture

A murine embryonic stem cell line (D3) transfected with an Oct4-GFP construct were used (phOCT3-EGFP1; provided by Wei Cui, Ph.D., Imperial College, London, UK). For this particular experiment these cells were used after several passages and splits, and thus did not show robust GFP activity under confocal or flow cytometry; thus immunostaining was necessary to visualize Oct4 expression. These cells were cultured in monolayer on 100 mm tissue culture plates coated with 0.67% gelatin in Dulbecco's modified Eagle's medium (DMEM) supplemented with 15% fetal bovine serum(FBS) (Hyclone, Logan, UT), 2 mM L-glutamine (Mediatech), 100 U/ml penicillin, 100 ug/ml streptomyocin, and 0.25 ug/ml amphotericin (Mediatech), 1× MEM nonessential amino acis solcuiotn (Mediatech), 0.1 mM 2-mercaptoethanol (FisherChecmical, Fairlawn, NJ), and 10^3^ U/ml leukemia inhibitory factor (LIF) (Chemicon Internation, Temecula, CA). Cells were passaged every 2–3 days prior to reaching 70% confluence.

### EB Formation and Culture

Undifferentiated embryonic stem cells were dissociated from monolayer culture using 0.05% trypsin-EDTA solution (Mediatech) to obtain a single cell suspension and added to AggreWells (Stem Cell Technologies) [62] six well plate inserts to form six thousand EBs of either 250 (1.5 million cells/ml) and 1000 (6 million cells/ml) cell per EB. EBs were allowed to form in the wells for 20 hours, at which point they were removed and transferred into a rotary culture at 60 RPM [63]. EBs were re-fed every 2 days, and 75% of the spent medium was replaced with fresh medium at each exchange. EBs were cultured in this manner for the entire 7 day culture period.

### Analysis of EB Size

EBs were harvested at various time points and fixed for 45 minutes in 10% formalin. EBs were imaged using bright field microscopy via a 4× objective on an EVOS microscope. Three representative images were taken for each sample. Images were analyzed by using threshold, watershed, and image particle detection operations in ImageJ. EB radius was derived by computing the cross sectional area, approximating the EB as a circle, and calculating the radius accordingly. The circularity of the EBs was calculated by fitting an ellipse to the area, and taking the ratio of the minor and major axes.

### Immunostaining and Confocal Microscopy

EBs were collected for staining and fixed in 10% formalin for 45 minutes. EBs were permeabilized for 30 minutes in 1.0% TritonX-100, re-fixed in formalin for 15 minutes, and blocked in blocking buffer (2% bovine serum albumin, 0.1% Tween-20 in PBS) for 3 hours. Samples were stained with a goat Oct4-antibody (Santa Cruz) overnight at 4°C. After three washes in blocking buffer, EBs were subsequently stained with a secondary donkey anti-goat Alexa Fluor 488 conjugated antibody (1∶200 Santa Cruz) for 4 hours. Staining with Alexa Flour 546 Phalloidin (1∶20 Molecular Probes) and Hoescht (1∶100) was performed concurrently for 25 minutes. Samples were washed, resuspended in blocking buffer, and imaged using a Zeiss LSM 510 Confocal Microscope using Ar, He, Ne and Chameleon lasers. A single image was taken at the top of the EB and at a depth of 25 µm into the EB. For each sample, 25 images were obtained.

### Pattern Analysis

Spatial patterns of Oct4 expression were classified into six different categories, random, inside-out, outside-in, connected, differentiated, or undifferentiated. For an image to be classified as undifferentiated, 90% or more of the cells in the image had to positively express Oct4. Conversely, for an image to be classified as differentiated only 10% of the cells could stain positive for Oct4. If the number of positive Oct4 cells fell in between these two levels, the pattern of differentiation was classified as either random, inside-out, outside-in, or connected (Fig. 4). Inside-out patterns were characterized by differentiation in the middle of the EB, and undifferentiated cells on the outside. Conversely, outside-in patterns had differentiation on the outside and undifferentiated cells in the middle. Connected patterns were defined as multiple distinct connected regions of cells of the same state. Random patterns were classified as no identifiable pattern. For each time point 25 confocal images were analyzed. Pattern matching was performed on the output from the model as well as the experimental confocal images. Two blinded observers were used to classify the experimental confocal images. In the case of the *in silico* results, metrics such as the total number of differentiated cells, and average number of distinct cell clusters were used to aid in classifying patterns, with a total of 73 1000-cell aggregates and 66 250-cell aggregates analyzed. A cluster was defined as two or more cells of the same type and clusters were identified throughout the entire 3D aggregate.

### Rules-Based Modeling

Rules-based modeling was carried out using probabilities to govern states changes. Linear, hyperbolic and hill-type probabilities have been previously examined in the context of stem cell differentiation and robustness and thus we used similar probability laws in this work [64]. All of these rules were designed to be functions only of the number of nearest neighbors to reduce complexity. For the “random” rule, a basal probability associated with the state change was set to 1% (Fig. 4A). In the case of the “positive feedback” rule, the differentiation probability was influenced according to equation 1:
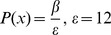
(1)where β represents the number of nodes connected in the differentiated state, normalized by the total possible number of neighboring nodes ε, which for a face-centered cubic or hexagonal close-packed spherical packing arrangement is 12 (Fig. 4B). In the case of the “competing feedback rule” rule the probability was determined according to equation 2:
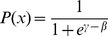
(2)where β represents the number of neighboring nodes in the differentiated state, and γ represents the number of neighboring nodes in the undifferentiated state (Fig. 4C). This function produces a similar sigmoidal shape as the Hill function, but does not require the inclusion of the additional hill coefficient.

### Computational Tools

Rules-based modeling was achieved using a Python language with the following freely available software packages: pyode, numpy, matplotlib, python imaging library (PIL) and vpython. Physical aggregation simulations of structure were performed using PyODE as the underlying physics engine. Results were plotted via the aid of numpy and matplotlib. The 2D aggregate slices were visualized using PIL. The 3D aggregate was visualized using vpython.

Simulations were run until all of the cells had changed state or 500 time steps had elapsed. Cells were allowed to make fate decisions ever time step according to the probabilities outlined in the Rules-Based modeling section. The time-step cutoff was arrived at by taking the average number of time steps the “random” rule simulations (as the “random” rule took the longest) took to finish. Unless otherwise noted, 10 simulations were run for each rule condition. Simulations were run on an Intel Core i7 X980 3.33 GHz CPU with 12.0 GB of RAM.

### Determining Growth Rate

After aggregate sizes were determined, the number of cells in a spheroid was approximated by first determining the volume of the spheroid based upon known EB radii. Next we calculated the volume of an average cell using our average cell diameter of 6.6+/−.3287 µm. By assuming a maximal close packed configuration for spheroids (.7408), the volume of the aggregate was adjusted to contain the cells. Cell numbers were then arrived at by dividing the adjusted aggregate volume by the volume of a single spheroid. To calculate the growth rate equation 3 was applied between discrete time points.
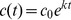
(3)This method produced growth rates over the first time course which closely matched the proliferation of mESC d3s in 2D. These growth rates could then be fit to equation 4 to determine doubling times which were used in the model for the different cell types.
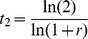
(4)


### Dynamic Modeling

Modeling of dynamic cell movement and vision was accomplished using custom C# code with the aid of XNA package for vector math and 3D visualization. Cells were modeled as rigid spheres connected by spring to depict cell-cell physical connections. A complete collision detection algorithm was used to resolve all possible collisions at each time step of the simulation.

Simulations were run for a period of 144 hours (6 days), until 99 percent of the cells had changed state or until forty thousand total cells existed in the model. Cells were allowed to change fate instantaneously. The kinetics of the simulations were fit to model growth curves, thus the probabilities were given different weights to assure pattern formation was observed. In the case of the random and positive feedback rules, no weights were applied to the rules. However, in the case of the competing feedback rule a 0.01 weight was applied. 10 simulations were run for each condition. Simulations were run in parallel using on an Intel Core i7 X980 3.33 GHz CPU with 12.0 GB of RAM.

### Model Assumptions

The static and dynamic models were created with several key assumptions. With regards to the probability rules themselves, it was assumed that the rules were functions of the information from immediate neighbors. The rule were derived from previous literature which examined similar probability functions [64]. Also it was assumed that the cells could be expressed in a binary state based on previous work modeling differentiation as an all or none response [52]. For the static model the following assumptions apply: first, the probability functions are applied every time step as the kinetics of pattern formation were not the focus of these models. Second, neighboring cells were assumed to have the same amount of influence over each other regardless of the number of neighbors. That means that in the case where a cell only has one connection, this connection still conveys the same weight as any given connection in the rest to the network for conveying information. The final assumption for this model was that the cells change fate instantaneously as kinetics was again not a focus.

For the dynamic model the following assumptions were used: first, it was assumed that the cells were not synchronized in terms of division. Second, cell division was symmetrical, meaning that cells only produced cells of the same type upon division. Third, cells were again assumed to have the same amount of influence over their neighbors regardless of the number of connections. Finally, the loss of pluripotency process was again modeled as an instantaneous transition.

#### Statistical analysis

All experiments were performed in triplicate and data was presented as the mean +/− the standard deviation. Significance was determined using a student's t-test with a significance level of p<0.05.

## Supporting Information

Figure S1 **Modeling spatial patterns of formation for 250-cell EBs.** (A, D, G) Pattern trajectories are shown for all three rules for 250 cell EBs plotted against a normalized time axis (τ) where the time step was divided by the total number of time steps required for the simulation to complete. (B, E, H) Representative “virtual sections” of aggregates over the course of a simulation. Cyan represents Oct4+ cells while blue signifies Oct4− cells. (C, F, I) Differences in the kinetics of modeled differentiation for all three rules. All scale bars are 25 µm.
(TIF)

Figure S2 **Initial aggregate structure has no effect on simulated pattern formation.** Trajectories from simulations run with varying different starting strucutres of the same size were compared to 10 simulations run on one structure to see if any differences in pattern formation emerged. For 250- and 1000-cell aggregates, the competing feedback (A, D), positive feedback (B, E) and random (C, F) rules were analyzed for variability. No observable differences were detected between different structures.
(TIF)

Figure S3 ***In silico*** **rule pattern trajectories.** Rule trajectories are composed of 3D traces with the undifferentiated cluster number (UCN), differentiated cluster number (DCN), plotted against a normalized time (τ) axis. Traces are plotted for both 1000 (A) and 250 (B) cell EBs for each of the three rules: competing feedback (green), positive feedback (red), and random (blue).
(TIF)

Figure S4 **Dynamic modeling does not alter network structure.** Control metrics for the dynamic cell simulations were plotted as functions of time: cell number, (A), radius (B), density (C), and circularity (D). Internal network parameters for the average connection length and average number of connections as a function of aggregate radius (E) behaved as expected. A representative trace for the growth of an EB starting at 50 cells shows a visual representation of the growing EB structrues (F).
(TIF)
