# Supplementary figures and images for: Spatial Pattern Dynamics of 3D Stem Cell Loss of Pluripotency via Rules-Based Computational Modeling
Source: PLoS Comput Biol. 2013 Mar 14;9(3):e1002952. doi: 10.1371/journal.pcbi.1002952 (PMC3597536; doi:10.1371/journal.pcbi.1002952)

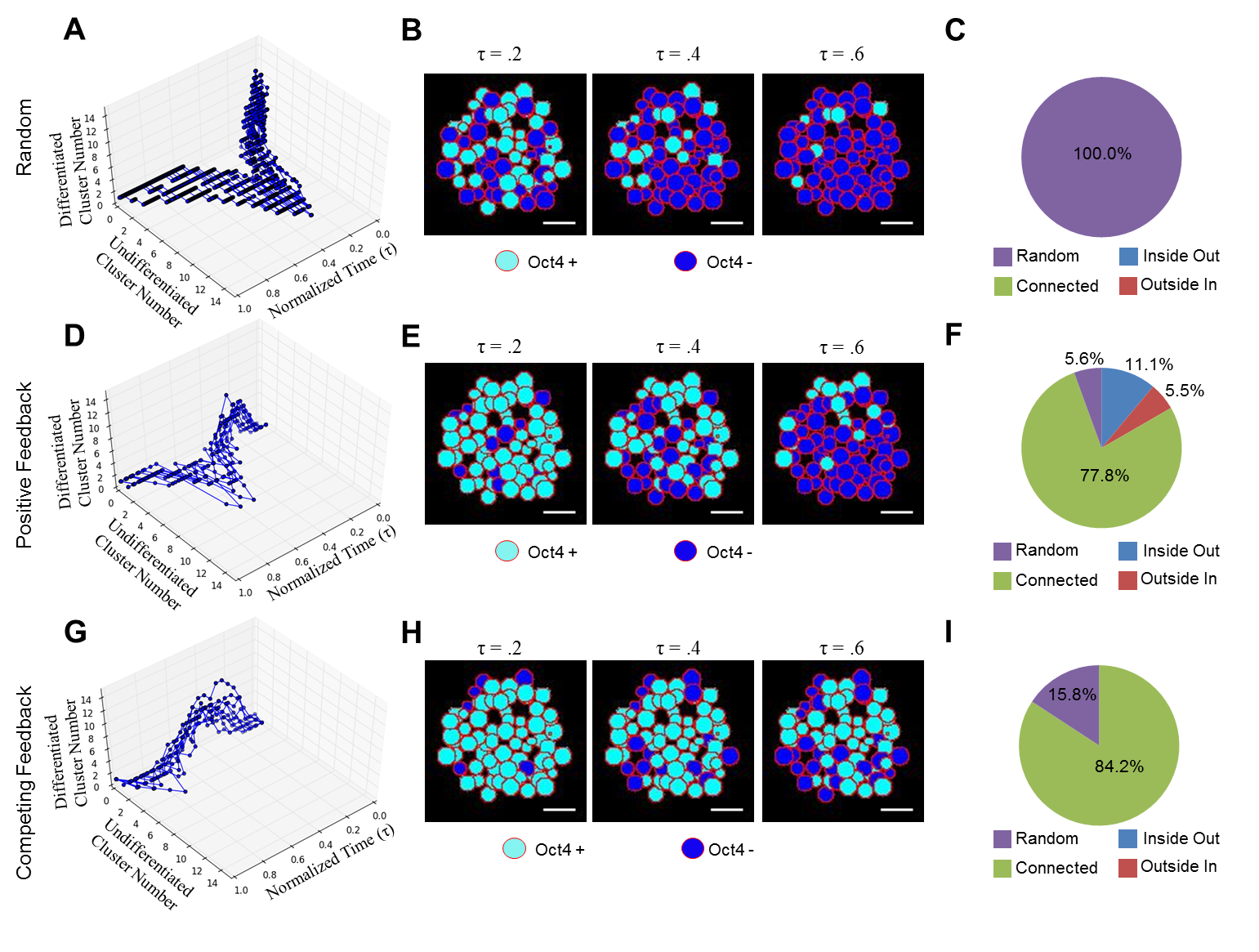

Supplement: Figure S1 — Modeling spatial patterns of formation for 250-cell EBs. (A, D, G) Pattern trajectories are shown for all three rules for 250 cell EBs plotted against a normalized time axis (τ) where the time step was divided by the total number of time steps required for the simulation to complete. (B, E, H) Representative “virtual sections” of aggregates over the course of a simulation. Cyan represents Oct4+ cells while blue signifies Oct4− cells. (C, F, I) Differences in the kinetics of modeled differentiation for all three rules. All scale bars are 25 µm. (TIF) [file pcbi.1002952.s001.tif]

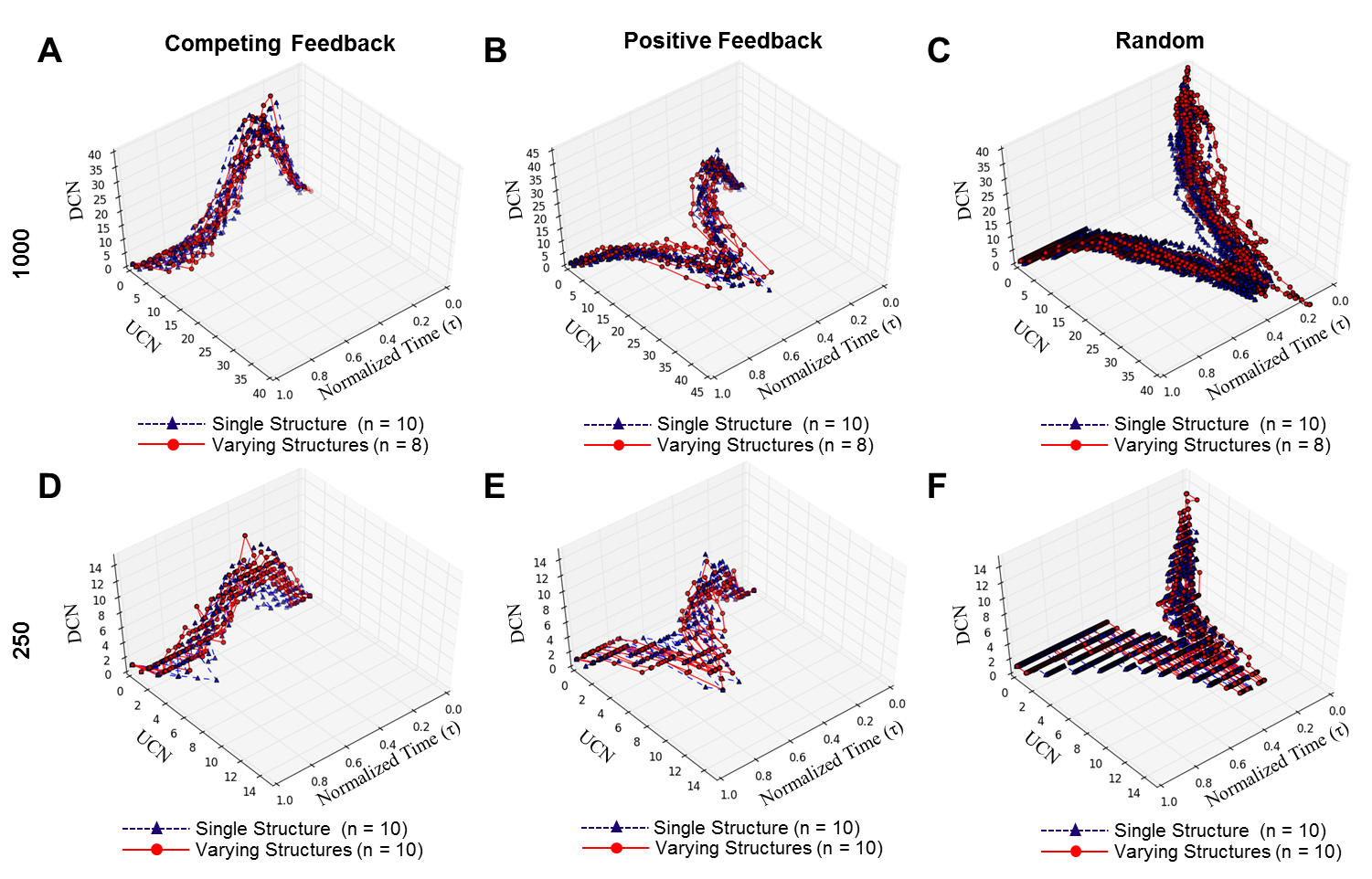

Supplement: Figure S2 — Initial aggregate structure has no effect on simulated pattern formation. Trajectories from simulations run with varying different starting strucutres of the same size were compared to 10 simulations run on one structure to see if any differences in pattern formation emerged. For 250- and 1000-cell aggregates, the competing feedback (A, D), positive feedback (B, E) and random (C, F) rules were analyzed for variability. No observable differences were detected between different structures. (TIF) [file pcbi.1002952.s002.tif]

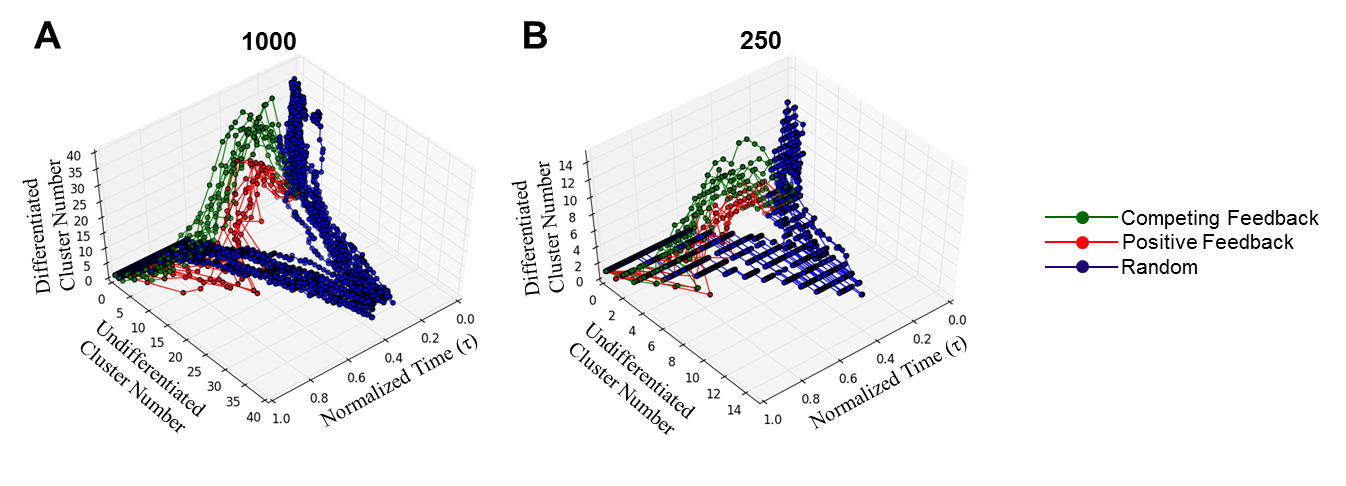

Supplement: Figure S3 — In silico rule pattern trajectories. Rule trajectories are composed of 3D traces with the undifferentiated cluster number (UCN), differentiated cluster number (DCN), plotted against a normalized time (τ) axis. Traces are plotted for both 1000 (A) and 250 (B) cell EBs for each of the three rules: competing feedback (green), positive feedback (red), and random (blue). (TIF) [file pcbi.1002952.s003.tif]

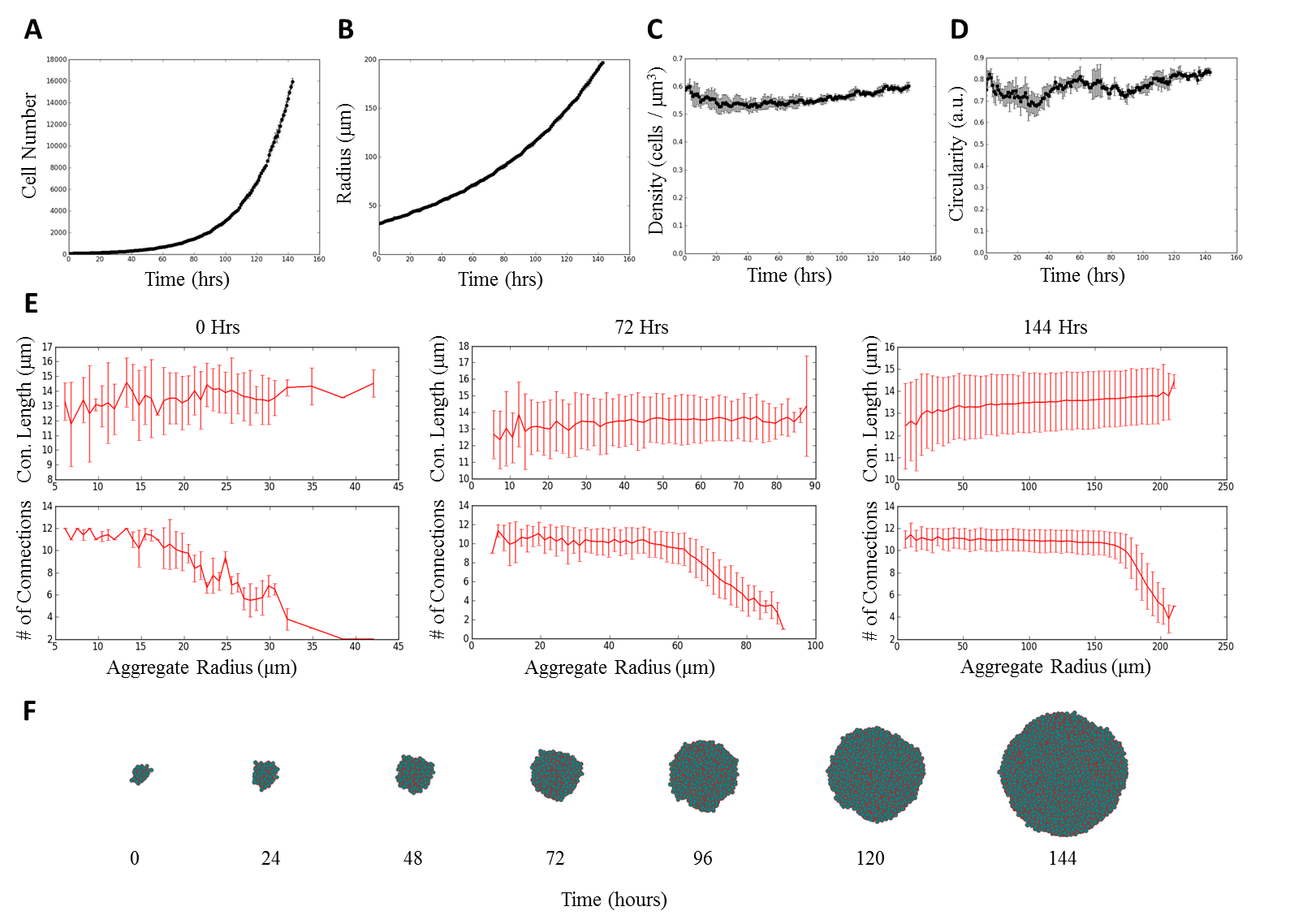

Supplement: Figure S4 — Dynamic modeling does not alter network structure. Control metrics for the dynamic cell simulations were plotted as functions of time: cell number, (A), radius (B), density (C), and circularity (D). Internal network parameters for the average connection length and average number of connections as a function of aggregate radius (E) behaved as expected. A representative trace for the growth of an EB starting at 50 cells shows a visual representation of the growing EB structrues (F). (TIF) [file pcbi.1002952.s004.tif]
